# Eukaryotic translation initiation factor eIF4G2 opens novel paths for protein synthesis in development, apoptosis and cell differentiation

**DOI:** 10.1111/cpr.13367

**Published:** 2022-12-22

**Authors:** Yudi Liu, Jiuwei Cui, Andrew R. Hoffman, Ji‐Fan Hu

**Affiliations:** ^1^ Key Laboratory of Organ Regeneration and Transplantation of Ministry of Education, Cancer Center, First Hospital Jilin University Changchun Jilin P.R. China; ^2^ Stanford University Medical School VA Palo Alto Health Care System Palo Alto California USA

## Abstract

Protein translation is a critical regulatory event involved in nearly all physiological and pathological processes. Eukaryotic translation initiation factors are dedicated to translation initiation, the most highly regulated stage of protein synthesis. Eukaryotic translation initiation factor 4G2 (eIF4G2, also called p97, NAT1 and DAP5), an eIF4G family member that lacks the binding sites for 5′ cap binding protein eIF4E, is widely considered to be a key factor for internal ribosome entry sites (IRESs)‐mediated cap‐independent translation. However, recent findings demonstrate that eIF4G2 also supports many other translation initiation pathways. In this review, we summarize the role of eIF4G2 in a variety of cap‐independent and ‐dependent translation initiation events. Additionally, we also update recent findings regarding the role of eIF4G2 in apoptosis, cell survival, cell differentiation and embryonic development. These studies reveal an emerging new picture of how eIF4G2 utilizes diverse translational mechanisms to regulate gene expression.

## Graphical abstract

Eukaryotic translation initiation factors 4G2 (eIF4G2) participates in diverse translation initiation, including translation driven by internal ribosome entry sites (IRESs), cap‐independent translation enhancers (CITEs), and N6‐methyladenosine (m6A), as well as in non‐canonical eIF4F‐independent cap‐dependent translation and canonical cap‐dependent scanning translation initiation. Through the translational mechanisms to regulate gene expression, eIF4G2 is significantly involved in apoptosis, cell survival, cell differentiation, and embryonic development processes.

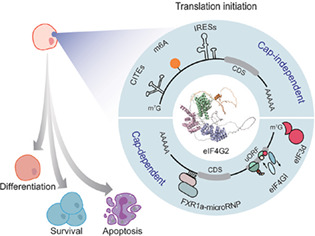

## 1 INTRODUCTION

In eukaryotic cells, the translation process can be divided into four main stages: initiation, elongation, termination, and ribosome recycling, with translation initiation being the time‐limiting and the most highly regulated stage.^1^, ^2^ At least 12 proteins, termed eukaryotic initiation factors (eIFs), are dedicated to translation initiation, recruiting the small ribosomal subunit (40 S) to the 5′ untranslated regions (UTRs) of mRNAs. The canonical cap‐dependent scanning translation initiation is based on the binding of eIF4F to the 7‐methylguanosine cap (m^7^G cap) at the 5′ end of a mRNA. eIF4GI plays a central role, acting as a multipurpose ribosome adapter,^3^ and bridging other eIFs, such as eIF4E, eIF4A, poly(A) binding protein (PABP) and eIF3, to enable ribosome recruitment. However, this canonical cap‐dependent translation initiation is blocked under conditions of cellular stress,^4^ tumorigenesis,^5^, ^6^ or viral infections.^7^ Thus, alternative modes of translation initiation that bypass the cap binding of eIF4F are required to maintain cell growth during cell stress. There are numerous non‐canonical mechanisms of initiation,^8^ which can be classified into two groups here for the sake of presentation, depending on whether 5′ cap binding is required. In group one, translation is cap‐dependent and eIF4F‐independent, and alternative proteins such as eIF3d bind to the 5′ cap.^9^ In group two, translation is cap‐independent, and translation may be driven by internal ribosome entry sites (IRESs),^10^ cap‐independent translation enhancers (CITEs),^11^ and N6‐methyladenosine (m6A).^12^

**FIGURE 1:**
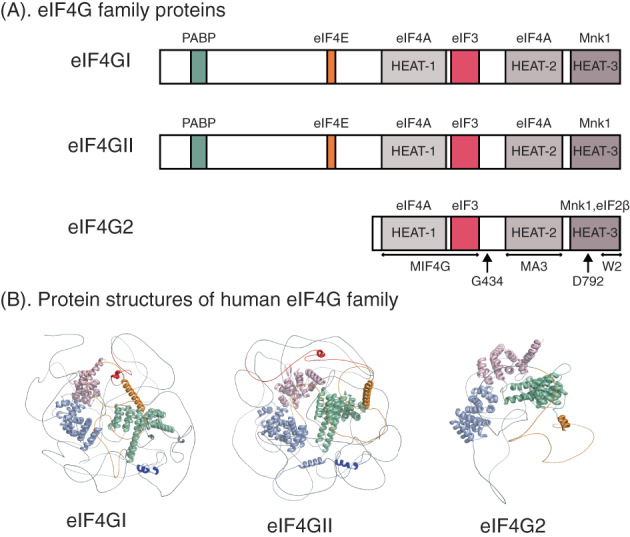
The structures of human eIF4G protein family. (A) The domain structures of the mammalian eIF4G family. eIF4G2 lacks the N‐terminal one‐third of eIF4GI and eIF4GII, which contains the binding sites for poly(A) binding protein (PABP) and eIF4E. Domains of interaction between eIF4G and proteins are shown in coloured boxes, and their interacting proteins are labelled above the domain boxes. Each isoform of eIF4G contains three HEAT domains, with the names listed under the box. Arrows: the cleavage sites of eIF4G2. The G434 site of eIF4G2 can be cleaved by viral protease 2A, and the D792 site by caspase. (B)Protein structures of human eIF4G family. The protein structure of each eIF4G family is predicted by Alphafold2 program.^115^, ^116^ The HEAT‐1, HEAT‐2, and HEAT‐3 domains are shown in green, purple, and pink, respectively. The interaction domains of PABP, eIF4E and eIF3 are shown in red, blue, and orange. eIF4G2 lacks the N‐terminal binding domains for PABP and eIF4E.

eIF4G2, also known as p97, NAT1 and DAP5, is a member of the eIF4G protein family, consisting of the major isoform eIF4GI (gene: *EIF4G1*), the least‐expressed isoform eIF4GII (gene: *EIF4G3*), and eIF4G2 (gene: *EIF4G2*) (Figure 1). eIF4G2 was identified simultaneously by four independent groups in 1997.^13^, ^14^, ^15^, ^16^ Shaughnessy et al. cloned mouse *Eif4g2* in an effort to isolate novel genes surrounding a common site of viral integration in myeloid leukaemia in BXH2 mice. They mapped human *EIF4G2* to a cluster of genes harbouring several unidentified tumour suppresser genes.^16^ In parallel, Imataka et al. cloned the *EIF4G2*/*p97* gene in order to identify genes encoding proteins with homology to the eIF4G family.^13^ Levy‐Strumpf et al. identified the full‐length *EIF4G2*/*DAP5* cDNA based on a dominant negative miniprotein, which was rescued by cDNA library transfections and apoptosis‐resistant cell selection.^15^ Finally, Yamanaka et al. identified *Eif4g2*/*Nat1* as a novel target for mRNA editing of Apobec‐1, which possesses RNA‐binding and cytidine deaminase activities.^14^

**FIGURE 2:**
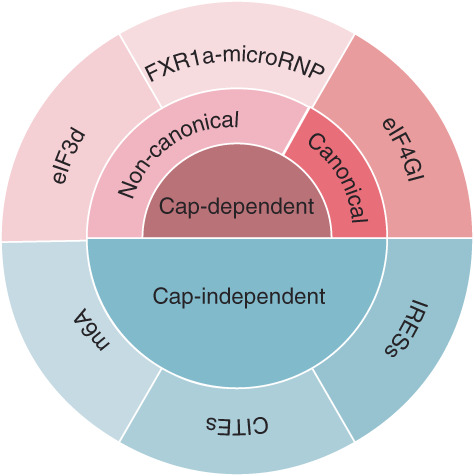
Summary of the eIF4G2‐involved translation initiation. eIF4G2 supports internal ribosome entry site (IRES)‐, cap‐independent translation enhancer (CITE)‐, and N6‐methyladenosine (m6A)‐mediated translation, and promotes non‐canonical cap‐dependent translation, in which alternative proteins, like eIF3d and PARN in FXR1a‐associated microRNP‐mediated translation initiation, bind m^7^G cap of mRNAs. At the same time, eIF4G2 also participates in the activation of the eIF4GI‐dependent canonical cap‐dependent translation.

eIF4GI and eIF4GII mainly participate in canonical cap‐dependent translation initiation. In contrast, eIF4G2 lacks the binding sites for cap binding protein eIF4E and for PABP.^13^, ^14^ Thus, early reports of eIF4G2 focused on its role in cap‐independent translation (Table 1). As the best understood mode of cap‐independent translation initiation, IRES‐driven translation was initially studied.^17^, ^18^, ^19^ Latter publications demonstrated that eIF4G2 supported other forms of cap‐independent translation including CITE‐^20^ and m6A‐driven^21^, ^22^, ^23^ translation initiation. More recent studies showed that eIF4G2 promoted non‐canonical^24^, ^25^ and even canonical cap‐dependent translation (Figure 2).^26^ In addition, eIF4G2 plays an important role in physiological processes including apoptosis, cell survival, cell differentiation, and embryonic development through activating the translation of critical proteins involved in these processes. We will review the structure of eIF4G2 and update its function in different modes of translation initiation, demonstrating how these findings have deepened our understanding of mechanisms of gene expression in eukaryotic cells.

**TABLE 1:** eIF4G2‐dependent mRNAs and circRNAs.

Name	References
Cap‐independent translation initiation	
Cellular IRESs	
Eukaryotic translation initiation factor (eIF) 4G2 (eIF4G2)	17,18,71,78,79,84
c‐Myc	18,78
X‐linked inhibitor of apoptosis (XIAP)	18,78
Apoptotic peptidase activating factor 1 (Apaf‐1)	71,78,79
Cellular inhibitor of apoptosis 1 (c‐IAP1)/ HIAP2	18,80,84
B‐cell lymphoma 2 (Bcl‐2)	71,85
Cyclin‐dependent kinase 1 (CDK1)	85
p53	19,20,75
High mobility group nucleosomal binding domain 3 (HMGN3)	93
Fibroblast growth factor 9 (FGF‐9)	20
CITEs	
Hypoxia‐inducible factor 1a (HIF‐1a)	20
p53	20
m6A	
Forkhead box protein O3 (FOXO3)	22
circ‐ZNF609	23
Cap‐dependent translation initiation	
Poly(rC)‐binding protein 2 (PCBP2)	91
Mitogen‐activated protein kinase kinase kinase 3 (Map3k3)	91
eIF3d	
c‐JUN	24
Matrix metalloproteinases (MMP1)	24
Cyclin‐dependent kinase 12 (CDK12)	24
CD101	25
CD103	25
FXR1a‐associated microRNP	
Myelin transcription factor 1 (Myt1)	100
Tumour necrosis factor α (TNFα)	100
eIF4GI	
Maf1	26
START domain containing 7 (Stard7)	26
Uncoupling protein 2 (UCP2)	26
Others	
Son of sevenless homologue 1 (Sos1)	114
Terminal uridylyl transferase 7 (TUT7)	92

Abbreviations: CITEs, cap‐independent translation enhancers; IRESs, internal ribosome entry sites; microRNP, microRNA‐protein complex; m6A, N6‐methyladenosine.

## 2 AN OVERVIEW OF TRANSLATION INITIATION IN EUKARYOTES

### 2.1 Canonical cap‐dependent translation initiation

**FIGURE 3:**
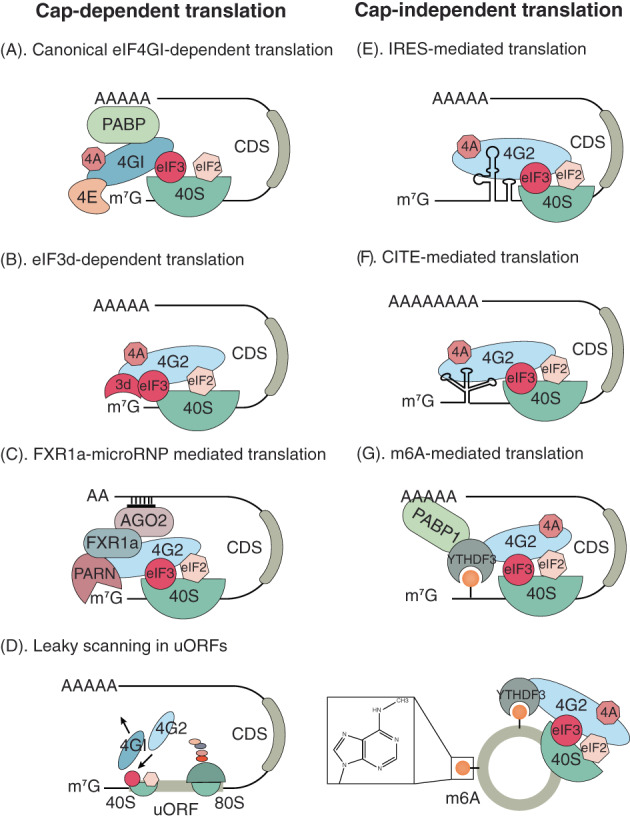
eIF4G2 is involved in both cap‐dependent and cap‐independent translation initiation. (A)The canonical eIF4GI‐dependent translation initiation. 40 S: the 40 S ribosomal subunit; CDS: the coding sequence; m^7^G: N7‐methylguanosine; 4GI: eIF4GI; 4A: eIF4A; PABP: poly(A) binding protein. eIF4GI binds to cap‐binding protein eIF4E, PABP, eIF4A and eIF3, recruiting the 40 S ribosomal subunit to initiate the translation. (B)The eIF3d‐dependent translation. 4G2: eIF4G2; 3d: eIF3d. eIF3d binds to the m^7^G cap of mRNA and eIF4G2 binds directly to eIF3d. Different from eIF4GI, eIF4G2 binds the eIF2β subunit. The eIF3d‐eIF4G2 complex activates the translation of some mRNAs. (C)The FXR1a‐associated microRNP‐mediated translation initiation. FXR1a: Fragile‐X‐mental‐retardation‐syndrome‐Related protein 1a. In quiescent conditions, poly(A) tails are shortened, avoiding binding to PABP. eIF4G2 is recruited to the 3′ UTRs via the interaction with microRNP containing AGO2 and FXR1a. The microRNP recruits cap‐binding protein PARN to initiate the translation. (D)eIF4G2 promotes leaky scanning through translated uORFs. uORFs: upstream open reading frames; 80 S: the 80 S ribosome. In the same uORF, eIF4GI can be dissociated from 43 S preinitiation complex (PIC) when the latter interferes with elongating 80 S ribosomes. In that case, eIF4G2 replaces eIF4GI in scanning complexes and resumes scanning afterwards. (E)The IRES‐mediated translation initiation. IRES: internal ribosome entry site. eIF4G2 binds to the IRES elements inherent in the 5′UTR of mRNAs or residing in the CDS. The 43 S PIC recruits the 40 S subunits to the regions of 5′UTR near the start codons of CDS, initiating the translation. (F)The CITE‐mediated translation initiation. CITE: cap‐independent translation enhancer. eIF4G2 binds to the CITE elements, recruiting 40 S subunits to the regions of 5′UTR near m^7^G cap. The 43 S preinitiation complex (PIC) scans from the 5′ end of mRNA for the start codon of CDS. (G)The m6A‐mediated mRNA translation. The m6A in the 5′UTR of mRNA recruits m6A reader YTHDF3, which binds eIF4G2 and PABP1 to initiate the translation process. Additionally, eIF4G2 and YTHDF3 recognize circRNAs via m6A, modulating their translation.

Canonical cap‐dependent scanning translation initiation^1^, ^2^ begins with the formation of a ternary complex (TC) of Met‐tRNAi, GTP and eIF2, which is composed of eIF2α, eIF2β and eIF2γ subunits. The TC then assembles with 40 S subunit, eIF1, eIF1A, eIF3 and eIF5 to form a 43 S preinitiation complex (PIC) (Figure 3A). eIF4F comprises the cap‐binding protein eIF4E, the RNA helicase eIF4A, which facilitates ribosomal recruitment by unwinding the 5′ cap‐proximal region of mRNA, and the large scaffolding protein eIF4G (both eIF4GI and eIF4GII), which binds eIF4A and eIF4E in the complex. eIF4G and eIF4B (eIF4H) enhance the helicase activity of eIF4A. eIF4G also has binding sites for eIF3, PABP, and kinases Mnk1/2, which phosphorylates eIF4E to stimulate translation. eIF4G engages PIC via interaction with eIF3, forming a circularized mRNA/RNA‐binding protein complex, in which the mRNA is activated by binding eIF4E at the 5′ cap and PABP at the 3′ poly(A) tail. Thus, the recruitment of PIC to the mRNA is ultimately facilitated by the cap–eIF4E–eIF4G–eIF3–40 S interaction. After it loads onto the mRNA, the PIC moves in the 3′ direction, scanning for a start codon within a suitable context. An optimum context is the consensus Kozak sequence, C(A/G)CCAUGG,^27^, ^28^ with a purine in −3 and guanosine in +4 positions (relative to the A of the AUG start codon, designated +1), that provides stable interactions with the PIC.^28^, ^29^, ^30^ Although AUG is the typical start codon, near‐cognate codons (e.g., CUG, GUG, and UUG, et al.) can also be used, albeit with varying efficiencies.^31^ Once base pairing between the Met‐tRNAi anticodon and the mRNA AUG codon is established, scanning arrests, eIF2·GTP is hydrolyzed, and conformational changes of PIC lead to dissociation of eIF1, eIF2·GDP, and eIF5 from PIC.^32^, ^33^ eIF1A remains 40 S bound, collaborating with eIF5B·GTP to mediate the joining of the large ribosomal subunit (60 S). After the release of eIF1A and eIF5B•GDP, the 80 S ribosome can enter the next elongation stage of protein synthesis.

### 2.2 Cap‐independent translation initiation

#### 2.2.1 Internal ribosome entry sites (IRESs)

IRESs are the RNA elements that recruit ribosomes to the internal region of mRNAs, rather than to the 5′ end, to initiate translation through multiple RNA–RNA and/or RNA–protein interactions.^10^, ^34^, ^35^ IRESs were initially identified in encephalomyocarditis virus (EMCV) and poliovirus (PV).^36^, ^37^ Subsequently, a large number of IRESs have been shown to exist in other viruses^38^, ^39^, ^40^ as well as in cellular mRNAs.^41^, ^42^, ^43^

Viral IRESs can be categorized into four major groups according to their structures, as well as their eIF and the IRES *trans*‐acting factor (ITAF) requirements.^10^, ^34^, ^35^ Generally, shorter IRESs with more compact and sophisticated RNA structures require fewer eIFs and ITAFs to initiate translation. Type I members, found only in enteroviruses (e.g., PV, Coxsackievirus B3 [CVB3]) and rhinoviruses,^37^, ^44^, ^45^ contain basic and flexible secondary structures which consist of short and long hairpins. Type II IRESs are found in EMCV and foot and mouth disease virus (FMDV).^36^, ^38^, ^46^ Although type II IRESs are structurally different from type I IRESs, both groups require multiple eIFs (e.g., eIF2, eIF3, eIF4A, and eIF4G).^47^, ^48^ Type I IRESs are able to recruit the 40 S subunit upstream of the coding region, where the ribosome scans the RNA in a 5′–3′ direction to find the downstream start codon.^48^ The major difference between type I and type II IRESs is that type II IRESs initiate translation directly at the 40 S subunit recruitment site without scanning step.^49^ Type III IRESs are folded into more sophisticated secondary and tertiary structures, through which they can directly interact with 40 S ribosome with the assistance of eIF2, eIF3, eIF5 and Met‐tRNAi to promote translation initiation.^50^ Type IV IRESs^51^ contain multiple stem‐loop structures including three pseudoknots, and they can also directly hijack the 40 S subunit. They do not require any eIFs or even TC. Instead, they begin translation from a non‐AUG start codon utilizing alanine‐tRNA (Ala‐tRNA) rather than Met‐tRNAi.^52^

Notably, IRESs are also found in cellular mRNAs.^8^, ^10^, ^41^, ^53^ A recent high throughput screening study indicated that IRESs were contained in an estimated 10% of mammalian mRNAs.^43^ As transcriptional products of RNA Pol II, all cellular mRNAs are supposed to have m^7^G caps and can bind the eIF4F complex to be translated by canonical cap‐dependent mechanism. However, the 5′ UTRs of putative IRES‐containing cellular mRNAs are usually GC‐rich, relative long, highly structured and may have several upstream open reading frames (uORFs),^54^ and these characteristics make it difficult for ribosomes to scan from the 5′ end. mRNAs with IRESs are translated in a cap‐dependent manner at low levels under normal conditions, and they can be translated robustly from IRESs under stress conditions when canonical cap‐dependent translation is compromised, including apoptosis, endoplasmic reticulum (ER) stress, hypoxia, mitosis, cell differentiation, and nutrient limitation. To accommodate this conversion of translational mechanisms, cellular IRESs contain fewer RNA structures than viral IRESs, and they can be composed of multiple short modules. Unlike viral IRESs, cellular IRESs are not conserved in sequence or structure, and thus it is difficult to classify them into groups. The existence of cellular IRESs still remains undetermined, mainly due to the unreliable systems used to validate IRES activity in certain cases.^55^ The role of cellular IRESs in translation has not been thoroughly elucidated and remains largely hypothetical. Similar to viral IRESs, cellular IRESs likely interact with 40 S subunits directly or through eIFs and ITAFs.

#### 2.2.2 Cap‐independent translation enhancers (CITEs)

CITEs, which were originally discovered in plant viruses^56^, ^57^ and have recently been found in animal cells,^11^ are another structural element supporting cap‐independent translation. CITEs may serve as an alternative to cellular IRESs. CITEs can reside in both 5′ and 3′ UTRs of mRNA, and they are required to expose the 5′ ends of mRNAs to scan for the start codon. On the other hand, IRESs are located only within the 5′ UTR of mRNA, and they direct the start codon within 5′ UTRs, not the 5′ ends, to the 40 S ribosome.

#### 2.2.3 N6‐methyladenosine (m6A)

m6A is the most abundant mRNA post‐transcriptional modification in eukaryotic cells. It modulates multiple stages of mRNA metabolism, which in turn regulates important biological processes, including cell differentiation and reprogramming.^12^, ^58^ The distribution of m6A along mRNAs is asymmetric, with enrichment in long exons, near stop codons and in 3′ UTRs.^59^, ^60^ While the extent of methylation in the 5′ UTR is relatively lower than elsewhere, it was reported that m6A in 5′ UTRs promoted cap‐independent translation in response to heat shock stress under the protection of m6A reader YTHDF2.^61^, ^62^

## 3 STRUCTURE AND MODIFICATIONS OF eIF4G2

eIF4G2 is a highly conserved, ubiquitously expressed protein, present in all Chordata and in many invertebrates.^13^, ^14^, ^15^, ^16^, ^63^, ^64^ eIF4G2 mRNA uses the GUG triplet as the translation initiation codon in mammals and nonmammalian vertebrates. In lower organisms, like *Halocynthia roretzi* and *Drosophila melanogaster*, eIF4G2 orthologs also use non‐AUG initiation codons, suggesting evolutionary conservation among species.^64^

### 3.1 Structure of eIF4G2

The 97‐kDa protein eIF4G2 is homologous to the C‐terminal two‐thirds of eIF4GI, which is produced by picornaviral proteolysis.^65^, ^66^ The N‐terminal portion of eIF4GI and eIF4GII that contain PABP and eIF4E binding regions are completely absent from eIF4G2^13^, ^14^ (Figure 1A). The greatest homology comparing eIF4G2 to eIF4GI is confined to the middle domain, termed MIF4G, which is 39% identical to human eIF4GI in amino acid sequence.^67^ This segment possesses a HEAT domain containing the eIF4A binding site and also has a binding site for eIF3. However, the affinity of the middle domain of eIF4G2 for eIF4A is 10‐fold lower than that of eIF4GI for eIF4A, implicating eIF4G2's weaker ability to stimulate eIF4A's RNA unwinding activity.^67^ The C‐terminal region of eIF4G2 contains two HEAT domains; the first domain is named MA3. The MA3 domain of eIF4G2 cannot bind eIF4A, in contrast to eIF4GI which has two binding sites for eIF4A. The second HEAT domain of both eIF4G2 and eIF4GI bind Mnk1 using two aromatic/acidic‐box (AA‐box; also called eIF5C or W2 domain) motifs. Interestingly, eIF4G2 but not eIF4GI binds to eIF2β through the AA‐box motifs.^68^, ^69^, ^70^, ^71^

### 3.2 Cleavage of eIF4G2 during apoptosis

eIF4G2 was identified by Kimchi et al. through a genetic screen aimed at isolating novel genes resistant to interferon‐γ‐induced programmed cell death (PCD) in Hela cells, and it was named death‐associated protein 5 (DAP5).^15^ The Kimchi group found that in response to activated Fas or p53, DAP5/p97 underwent proteolytic cleavage, giving rise to an 86‐kDa fragment (DAP5/p86) devoid of the C terminus. Using a panel of protease inhibitors, the cleavage of DAP5/p97 was ascribed to caspases. Cleavage site mutations revealed that DAP5/p97 was cleaved at a single caspase cleavage site at position 792 (Figure 1A).^17^ In line with the biochemical study, the group crystallized the C‐terminal domain of DAP5/p97, demonstrating that the cleavage of DAP5/p97 removed the third HEAT domain. As a result, DAP5/p86 might lose its ability to bind Mnk1 and eIF2β.^70^

### 3.3 Modifications of eIF4G2 by Coxsackievirus B3

CVB3, a positive single‐stranded RNA virus, is a primary cause of viral myocarditis^72^ and is associated with dilated cardiomyopathy and heart failure.^73^ Although an early study showed that the cleavage of eIF4G2 observed during CVB3 infection could not be attributed to CVB3 protease 2A or 3C,^74^ a more recent study^75^ demonstrated that eIF4G2 was cleaved by protease 2A but not 3C at amino‐acid reside G434 (Figure 1A), generating a 45‐kDa N‐terminal fragment (eIF4G2‐N) and a 52‐kDa C‐terminal fragment (eIF4G2‐C). The eIF4G2‐N partially translocated to the nucleus, and compared with the full‐length eIF4G2, it maintained IRES‐driven translation of p53, but not B‐cell lymphoma 2 (Bcl‐2). Further, eIF4G2‐N expression induced more viral capsid protein 1 as well as a higher viral titre during the late phase of infection. In contrast, eIF4G2‐C remained primarily in the cytoplasm where it inhibited cap‐dependent translation and promoted cap‐independent translation indirectly. Taken together, the truncated fragments led to viral replication and viral‐induced apoptosis, and thus promoted CVB3 progeny release.

## 4 THE ROLE OF eIF4G2 IN CAP‐INDEPENDENT TRANSLATION

### 4.1 eIF4G2 and IRES‐mediated translation.

#### 4.1.1 eIF4G2 and viral IRESs

For viral IRESs, eIF4G2, but not the full‐length of eIF4GI, facilitated the initial round of translation of CVB3 RNA.^76^ As mentioned above, CVB3 RNA contains type I IRES elements, which require the C‐terminal fragment of eIF4GI cleaved by CVB3 protease 2A. However, protease 2A, and thus, C‐terminal eIF4GI, is absent in the first round of viral RNA translation. Using the CVB3 model, it was proposed that eIF4G2 supported basal levels of translation before the cleavage of eIF4GI. Subsequently, the cleaved C‐terminal eIF4GI promoted protein synthesis from IRESs. eIF4G2 was only required in type I IRESs, but not in type II or type III.

#### 4.1.2 eIF4G2 and cellular IRESs

##### eIF4G2/p97 and eIF4G2/p86 in translation

For cellular IRESs, early studies suggested that eIF4G2 acted as a general repressor of protein synthesis under normal conditions.^13^, ^14^ When co‐transfected into HeLa or COS7 cells along with a bicistronic reporter plasmid, eIF4G2 overexpression decreased both cap‐dependent and cap‐independent translation from EMCV IRESs. Furthermore, overexpression of eIF4G2 in S2‐6 cells reduced overall translation as measured by [^35^ S] methionine incorporation. The repression of eIF4G2 was presumably caused by sequestering eIF3 and eIF4AI into inactive complexes. Another study showed no change in global translation or in activities of the IRESs of EMCV, c‐Myc and eIF4G2 itself when eIF4G2 was knocked out in mouse ESCs.^77^

Due to the structural differences, eIF4G2 failed to bind eIF4E,^13^, ^14^ an essential step for cap‐dependent translation. eIF4G2 has been studied with a focus on IRES‐driven translation (Figure 3E). Coincidently, while global protein synthesis was repressed in apoptosis, the translation rate of eIF4G2 was maintained, and many apoptosis‐associated proteins continued being translated through IRES elements. Thus, eIF4G2 mRNA itself was studied as a target of IRES‐mediated translation in apoptosis. The 5′ UTR of eIF4G2 mRNA encompasses some of the characteristics of IRES elements, as it contains two relatively long polypyrimidine‐rich tracts.^15^ Based on a bicistronic system in a rabbit reticulocyte lysate (RRL) ‐based cell‐free system, Henis‐Korenblit et al. suggested that eIF4G2 supported its own translation preferentially via the eIF4G2 IRES, providing a positive auto‐regulatory loop. Notably, the caspase‐cleaved eIF4G2/p86 form was more potent than eIF4G2/p97.^17^ A second study from this group showed that eIF4G2/p86 enhanced translation through the IRESs of eIF4G2, c‐Myc, X‐linked inhibitor of apoptosis (XIAP), and apoptotic peptidase activating factor 1 (Apaf‐1) using a bicistronic system in 293 T cells.^78^ Similarly, Holcík and colleagues showed that eIF4G2/p86, but not eIF4G2/p97, enhanced translation mediated by the cellular inhibitor of apoptosis 1 (c‐IAP1) /HIAP2, eIF4G2 and Apaf‐1 IRES elements based on a bicistronic system in 293 T cells. In contrast, no effect of eIF4G2/p86 on the translation from XIAP or c‐Myc IRES elements was observed.^79^, ^80^ Collectively, these results indicated that eIF4G2/p97 was activated by caspase cleavage, and the resulting DAP5/p86 form acted as a specific eIF for cellular IRESs, especially under conditions of cellular stress.^63^, ^81^, ^82^

However, these results have been challenged by several studies, which showed that the full‐length eIF4G2/p97 could positively promote protein synthesis in vivo and in vitro, even at steady‐state growth conditions. Using a HeLa‐based cell‐free translation system biochemically depleted of eIF4G, Hundsdoerfer et al. demonstrated that exogenous full‐length eIF4G2 strongly promoted the translation of ApppG‐capped hairpin‐containing reporter mRNAs from the eIF4G2, c‐Myc, c‐IAP1, and XIAP IRESs, but not cap‐ or HCV IRES‐dependent translation.^18^ Moreover, eIF4G2/p97 was found, in actively growing unstressed human cells, to be associated with polysomes, implying that it promoted translation under normal growth conditions, whereas eIF4G2/p86 was not detectable in polysomes in apoptotic cells.^83^ Another study showed that activation of eIF4G2 and c‐IAP1 IRESs required eIF4G2/p97 during ER stress induced by tunicamycin treatment. When co‐transfected with bicistronic plasmids in 293 T cells, overexpression of eIF4G2/p97 enhanced the activity of the eIF4G2 IRES, but not c‐IAP1 IRES. In contrast to the previous study, a siRNA‐mediated knockdown of DAP5/p97 failed to affect overall translation.^84^ In addition, by introducing capped bicistronic mRNAs in unstressed eIF4G2/p97‐depleted HeLa cells, Marash et al. established that eIF4G2/p97 supported translation of Bcl‐2 and cyclin‐dependent kinase (CDK) 1 in a cap‐independent manner. And eIF4G2/p97 knockdown did not affect global cap‐dependent mRNA translation.^85^ Although these results were somewhat inconsistent, they identified eIF4G2/p97 as an activator of translation from IRESs in the absence of proteolytic processing in both stressed and unstressed cells.

##### eIF4G2 and p53 mRNA translation

Although the majority of cellular IRES elements are located in the 5′UTR of mRNAs, a few IRESs reside in the coding region.^86^, ^87^ p53 mRNA contains two IRESs, with the first one in the 5′UTR, driving the translation of full‐length p53, and the second within the coding region, supporting the translation of N‐terminal truncated Δ40p53 isoform.^88^, ^89^, ^90^ eIF4G2 was shown to promote the translation of both isoforms of p53 from two IRESs. siRNA‐mediated knockdown of eIF4G2 reduced levels of endogenous and ectopically expressed p53 and Δ40p53 under stress conditions, with a greater effect on the latter, whereas upon eIF4G2 depletion in unstressed cells, only translation from the second IRES was selectively inhibited.^19^ These results suggest that in stress conditions where cap‐dependent translation was compromised, eIF4G2 drove the translation of both isoforms, but it only supported Δ40p53 translation from IRES in unstressed cells, in which full‐length p53 was translated through canonical cap‐dependent mechanism.

##### The regulation of eIF4G2 translation

Translation of eIF4G2 is regulated by mRNA‐binding proteins at the posttranscriptional level. Poly(rC)‐binding protein 2 (hnRNP E2/PCBP2) isoform f, which is an RNA‐binding protein in mammalian cells, inhibited the translation of eIF4G2 in vitro and in cultured cells via binding the polypyrimidine sequence within the 5′ UTR of eIF4G2 mRNA. Furthermore, eIF4G2 also supported the translation of a luciferase reporter carrying the 5′ UTR of PCBP2 as well as the endogenous PCBP2 protein. Therefore, eIF4G2 and PCBP2 mutually regulated one another's translation through a feedback loop.^91^

##### Mechanisms of eIF4G2‐mediated IRES‐driven translation

Although the above observations provided evidence that eIF4G2 was actively involved in viral and cellular IRES‐mediated translation initiation, the specific mechanisms remained unclear. First, assays were performed to determine whether eIF4G2 bound to target mRNAs directly or through the interaction with factors such as ITAFs. Immunoprecipitation‐based methods were used in several studies to identify mRNA transcripts physically interacting with eIF4G2, either directly or indirectly.^19^, ^84^, ^85^, ^92^ CDK1 was identified and validated as a translational target of eIF4G2 through this method.^85^ One study showed that eIF4G2/p97 supported the activity of the eIF4G2 IRES, but not the HIAP2 IRES. Co‐precipitation of FLAG‐tagged eIF4G2/p97 and endogenous mRNAs was performed, and eIF4G2/p97 was found to bind to both eIF4G2 and HIAP2 mRNAs.^84^ Yoffe et al., used an electrophoretic mobility shift assay (EMSA) to show that eIF4G2 supported the translation of high mobility group nucleosomal binding domain 3 (*HMGN3*) mRNA from IRES by directly binding to the first half of its 5′ UTR.^93^ Using an EMSA in vitro and immunoprecipitation method in cells transfected with bicistronic RNA carrying the first and second p53 IRESs, eIF4G2 was shown to bind specifically and directly to p53 mRNA, and more specifically, to all its two IRES elements. Although eIF4G2 bound both p53 IRESs with similar affinity, it had a greater effect on the second IRES, and it did not drive translation from the first IRES in unstressed cells.^19^ These observations suggest that in addition to mRNA binding, other factors also regulate the translation from IRESs. At certain parts of the cell cycle or under some stress conditions, the loss of IRES‐dependent translation may be caused by the inaccessibility of factors like ITAFs that can promote translation, or, alternatively, by the presence of repressors that inhibit the activity of IRESs and in turn, inhibit translation. Second, the canonical eIFs involved were also studied to further clarify the mechanism of cellular IRES‐directed translation initiation. eIF4G2 co‐sedimented with eIF2 in the 40 S ribosomal fraction in a growth factor dependent manner. Furthermore, by employing eIF4G2 E862K and N86A mutants, which were unable to bind eIF2β and eIF4AI respectively, it was observed that eIF4G2‐eIF2β and eIF4G2‐eIF4AI interactions were all required for IRES‐driven translation of Bcl‐2, Apaf1 and eIF4G2 mRNAs. In addition, eIF4AI helicase activity was also required. These results suggest that the eIF4G2‐eIF4AI‐eIF2β complex bound cellular IRESs to recruit the TC and ribosome into proximity of the initiation codon.^71^

### 4.2 eIF4G2 and CITE‐mediated translation

A more recent publication, based on a fluorescence anisotropy‐based equilibrium binding assay employing purified eIF4G2 protein and uncapped RNA oligonucleotides, showed that eIF4G2 bound directly to the 5′UTRs of hypoxia‐inducible factor 1a (HIF‐1a), fibroblast growth factor 9 (FGF‐9), and p53_A_ and p53_B_ mRNAs in a completely cap‐independent manner. eIF4G2 activated cap‐independent translation of the four UTR‐Luc mRNAs with nonfunctional cap analogues in an RRL translation system. By inserting a stable and scanning‐inhibiting hairpin at the 5′ UTR of ApppG‐capped UTR‐Luc mRNAs, it was shown that the accessibility of the 5′ end of HIF‐1a and p53_A_ mRNAs was required for this activity. These observations indicate that FGF‐9 and p53_B_ mRNAs were translated via IRES‐like mechanisms, whereas the translation of HIF‐1a and p53_A_ mRNAs required an accessible 5′ end although it was cap‐independent. Thus, HIF‐1a and p53A mRNAs employed a CITE‐like mechanism (Figure 3F).^20^

### 4.3 eIF4G2 in m6A‐mediated translation

In RAW264.7 cells, eIF4G2 enhanced the translation of transcription corepressor forkhead box protein O3 (FOXO3) by synergistically interacting with PABP1 and YTHDF3, an m6A reader of the YTHDF family and binding to the translation initiation region of FOXO3 mRNA. The depletion of eIF4G2, YTHDF3, or PABP1 reduced FOXO3 protein levels (Figure 3G).^22^

Unlike mRNA, circRNA does not have a free m6A‐modified 5′ UTR or a 3′ poly (A) tail that can bind PABP. However, circRNAs contain extensive m6A modifications and translation initiation sites, and many of them are associated with polysomes, suggesting that some endogenous circRNAs likely function as mRNAs. eIF4G2 was found to cooperate with YTHDF3 to promote translation from an artificial circRNA that encoded GFP. Consistent with previous findings,^61^ this study demonstrated that eIF3a was involved in the m6A‐driven translation.^21^ A more recent study has also indicated that eIF4G2 and YTHDF3 recognized endogenous circ‐ZNF609 via m6A, thereby modulating its translation. However, the translation of circ‐ZNF609 did not rely on eIF3a or eIF3b (Figure 3G).^23^

While it has recently been shown that many short ORFs (sORFs) of long non‐coding RNAs (lncRNAs) also encoded small peptides, the specific translation mechanisms remain largely unknown.^94^, ^95^ Although lncRNAs contain 5′ cap and 3′ poly(A) tails, it was found that eIF4E that was phosphorylated by MST1 interacted weakly with the 5′ cap and enhanced the translation of lncRNAs,^96^ suggesting the possibility that eIF4G2 participates in lncRNA small peptide translation.

## 5 THE ROLE OF eIF4G2 IN NON‐CANONICAL CAP‐DEPENDENT TRANSLATION

While eIF4G2 has been studied primarily in cap‐independent translation initiation, especially in IRES‐mediated translation, some reports suggest that eIF4G2 also participates in cap‐dependent translation.^68^, ^91^ Overexpression of eIF4G2 increased translation of cap‐dependent reporter mRNA in T98G cells. Conversely, depletion of eIF4G2 by RNA interference reduced global translation as well as reporter mRNA translation.^68^ Moreover, it was demonstrated that eIF4G2 supported the translation of reporter mRNAs containing 5′ UTRs from PCBP2 and mouse mitogen‐activated protein kinase kinase kinase 3 (Map3k3) in a cap‐dependent manner.^91^

More recently, several studies have focused on the specific mechanism of eIF4G2 in cap‐dependent translation. Schneider's group showed that eIF4G2 complexed with eIF3d to promote non‐canonical, cap‐dependent, eIF4E‐independent translation of approximately 20% of mRNAs in mammalian cells. Genome‐wide transcriptomic and translatomic analyses demonstrated that highly eIF4G2‐dependent mRNAs were enriched in cell survival, motility, DNA repair and translation initiation pathways. However, few of these mRNAs contained IRESs. Previously, eIF3d was identified as an mRNA m^7^G cap‐binding protein for translation of c‐Jun mRNA, regulatory‐associated protein of mTOR (Raptor) mRNA, and La‐related protein 1 (Larp1) mRNA.^97^, ^98^, ^99^ This work, based on mass spectrometry and crosslinking studies, showed that eIF4G2 is bound directly and strongly with eIF3d. The eIF4G2‐eIF3d complex supported cap‐dependent translation of mRNAs encoding c‐JUN, matrix metalloproteinases (MMP1), and CDK12^24^ (Figure 3B). Silencing of eIF4G2 completely inhibited protein levels of some identified eIF4G2‐dependent mRNAs, including ETS1, OSMR, L‐Myc, ITGV, p53‐BP1, and MMP1, while this silencing moderately reduced the levels of EFGFR and SERPINE2. The varying extent of eIF4G2‐dependence among these mRNAs suggests that certain mRNAs could employ either eIF4E‐eIF4GI or eIF4G2‐eIF3d complexes, or even both complexes equally for translation under some physiological conditions.

The same group also found that the eIF4G2‐eIF3d complex activated the translation of a set of Treg cell‐associated mRNAs in a cap‐dependent, eIF4E/mTOR complex 1 (mTORC1)‐independent manner. Generation of in vitro‐induced Treg (iTreg) cells required mTORC1 inhibition, and repressed mTOR activity induced hypo‐phosphorylation of 4EBPs, which in turn blocked the eIF4E‐eIF4G interaction, inhibiting canonical translation and global protein synthesis. In contrast to the reduced levels of eIF4GI, eIF4G2 and eIF3d levels strongly increased in differentiated iTreg cells. Using in vitro and in vivo monocistronic and bicistronic luciferase assays in human 293 T cells, it was shown that translation of mRNAs containing CD101 and CD103 5′ UTRs was cap‐, eIF4G2‐, and eIF3d‐dependent (Figure 3B). Interestingly, translation from the 5′ UTR of *FOXP3*, whose protein is expressed in both uncommitted activated CD4^+^ T cells and in iTreg cells, required both eIF4E and eIF4G2, suggesting a dual usage of both eIF4E‐eIF4GI and eIF4G2‐eIF3d mechanisms.^25^

eIF4G2 can also mediate non‐canonical, cap‐dependent translation during quiescent conditions, including in quiescent (G0) human THP1 cells and immature *Xenopus laevis* oocytes, where canonical eIF4E‐ and PABP‐dependent translation is compromised due to poly(A) tails shortening (shorter than a PABP site) and low mTOR kinase activity. Shortened poly(A) tails are caused by increased activity of Poly(A) ribonuclease (PARN), leading to less binding to PABP that is required in canonical translation. eIF4G2 was shown to be recruited to the 3′ UTRs via an interaction with microRNPs (microRNA‐protein complexes), which contained an Argonaute family member protein AGO2, and an RNA binding protein, Fragile‐X‐mental‐retardation‐syndrome‐Related protein 1a (FXR1a). The microRNPs recruited PARN, which recognized the m^7^G cap to support eIF4G2‐dependent translation of FXR1a‐microRNP associated myelin transcription factor 1 (Myt1) and tumour necrosis factor α (TNFα) mRNAs (Figure 3C).^100^, ^101^

## 6 THE ROLE OF eIF4G2 IN CANONICAL CAP‐DEPENDENT TRANSLATION

uORFs are short ORFs located in the 5′ UTRs of eukaryotic mRNAs, with start codons residing upstream of start codons of the main protein‐coding sequence (CDS). uORFs regulate the translation initiation of CDS by sequestering PIC. Thus, the majority of uORFs function as repressors of downstream translation. When a 43 S PIC scans along the 5′ UTR and encounters a start codon of a uORF (uAUG) in a poor context, it will bypass the suboptimal uAUG by leaky scanning and continue to scan for the next start codon. If a uORF is recognized and translated, the elongating 80 S ribosome may be stalled by the peptide encoded by the uORF at or near the uORF stop codon, creating a ‘roadblock’ to additional scanning PICs and triggering nonsense‐mediated decay (NMD). After termination, it is possible that both 40 S and 60 S subunits are released; alternatively, the 40 S subunits may remain on the mRNA, resume scanning, and reinitiate translation of the main CDS.^102^, ^103^, ^104^, ^105^ Increased length of the uORFs or stable RNA secondary structures of uORFs were shown to decrease rescanning and reinitiation efficiency,^1^ suggesting that the duration of uORF translation was crucial. This led to the idea that eIF–ribosome interactions might enhance initiation at uAUG and in turn, promote rescanning.

Unlike the eIF4G2‐involved cap‐dependent translation mentioned above, which was eIF4E‐independent and used alternative factors to bind the 5′ cap when the canonical translation was compromised, one study showed that, under normal conditions, eIF4G2 promoted leaky scanning through translated uORFs, the translation of which was canonical cap‐ and eIF4F‐dependent (Figure 3D). Many eIF4G2‐dependent mRNAs contain uORFs, and a subset of them require eIF4G2 for leaky scanning, including Maf1, START domain containing 7 (Stard7), and uncoupling protein 2 (UCP2) mRNAs. As the proposed model demonstrates, after cap recognition and eIF4GI‐mediated 40 S subunit recruitment and scanning, eIF4GI can be dissociated from the 43 S PIC scanning complexes when the latter interfered with elongating 80 S ribosomes engaged with the same translated uORF. Then eIF4G2 can replace eIF4GI in the scanning complexes and resume scanning afterwards. A previous study suggested that as a scanning ribosome moved downstream, the stability of its interaction with eIF4GI decreased.^106^ Thus, this mechanism was crucial for the translation of mRNAs with long 5′UTRs and highly frequent uORFs. For eIF4G2 mRNA targets without uORFs, especially those with long 5′UTRs, the model suggests that eIF4G2 would rescue scanning if eIF4GI was released from scanning complexes on its way to the uORFs because of obstacles such as secondary structures or RNA‐binding proteins. It appears that higher eukaryotes possess an accessory scanning complex to rescue scanning when the principal method failed.^26^

In contrast, a recent study showed that eIF4G2 negatively regulated the translation of G_4_C_2_ repeat‐containing RNA, whose translation was regulated by a uORF and was cap‐independent, by recruiting ribosomes to the uAUG and initiating translation. In this model, the translation of uORF was cap‐independent, and the involvement of eIF4G2 could not promote the translation of downstream ORF.^107^

## 7 FUNCTIONS OF eIF4G2

### 7.1 The role of eIF4G2 in apoptosis

**FIGURE 4:**
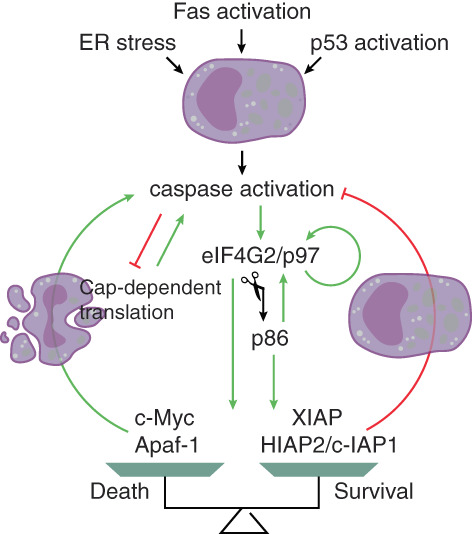
Regulation of apoptosis by eIF4G2‐initiated translation. The positive regulatory events of apoptosis are shown in green, and the negative events are shown in red. When cells are stimulated by activated Fas or p53, or prolonged ER stress, the caspases are activated, leading to inhibition of cap‐dependent translation, which in turn, promotes caspase cascade and cell death. The level of eIF4G2 is maintained, and eIF4G2 can be cleaved by caspases, giving rise to an 86‐kDa fragment. Both p97 and p86 promote the eIF4G2‐initiated translation, and support translation of pro‐apoptosis proteins like c‐Myc and Apaf‐1, and of anti‐apoptosis proteins such as XIAP and HIAP2/c‐IAP1. Using this strategy, eIF4G2 regulates the balance between cell death and survival.

eIF4G2 has been widely studied as a crucial factor involved in apoptosis and cell stress (Figure 4). eIF4G2 is constitutively expressed in cells under normal conditions. Despite the general translational block seen in stressed cells, eIF4G2 was preferentially and continuously translated during apoptosis. Furthermore, under apoptotic conditions, eIF4G2/p97 was converted to eIF4G2/p86 by caspase cleavage. Both p97 and p86 supported their own translation via the IRES element within the 5′UTR, providing a positive feedback loop.^17^, ^83^ eIF4G2 was diffusely localized in the cytoplasm in normal cells, and it moved to stress granules in response to oxidative stress or heat shock.^83^ In addition to eIF4G2, a subset of mRNAs also bypassed the global translational repression and selectively maintained their translation through cap‐independent mechanisms. While the results were inconsistent, it was shown that p97 and/or p86 activated IRES‐dependent translation of both proapoptotic proteins, such as c‐Myc and Apaf‐1,^18^, ^78^, ^79^ which enhanced the death cascade in a positive feedback loop, and of antiapoptotic proteins including XIAP and c‐IAP1/HIAP2,^78^, ^79^ which generated negative feedback to prevent cell death in the presence of stress triggers. Prolonged ER stress resulted in the induction of apoptosis. During ER stress, p97 supported its own translation from IRES, while c‐IAP1/HIAP2 IRES could only be activated by p86 but not p97.^80^, ^84^ Therefore, in conditions of cellular stress and apoptosis, eIF4G2 acts as a translational activator that regulates translation of specific mRNAs mediated by IRESs in both caspase‐dependent and caspase‐independent manners. It plays a complex role in apoptosis, fine‐tuning cell fate by balancing positive and negative mediators.

### 7.2 The role of eIF4G2 in cell survival

eIF4G2 promotes proliferation and survival of many unstressed mammalian cells. During mitosis, there is a ~35% reduction in global translation.^108^ However, when cells were arrested during mitosis by incubation with taxol, the level of eIF4G2 remained unchanged.^85^ Depletion of eIF4G2 from cells resulted in a substantial loss of cell viability.^68^, ^84^, ^85^ One study showed that knockdown of eIF4G2 by RNA interference inhibited cell proliferation in many mammalian cell lines, in correlation with a marked increase in the levels of cell cycle inhibitory protein p27/Kip1 and a decrease in CDK2 kinase activity.^68^ Another study found that eIF4G2 knockdown induced cell death in a caspase‐dependent way, in particular during the mitotic phase.^85^ These authors screened two eIF4G2 translation targets, Bcl‐2 and CDK1, which are pro‐survival proteins during mitosis. CDK1 was responsible for phosphorylation of CDK1 substrates, such as MPM‐2 and an M phase‐specific inhibitor of apoptosis survivin. Knockdown of eIF4G2 abrogated the IRES‐driven translation of Bcl‐2 and CDK1, and consequently reduced MPM‐2 reactivity and the steady‐state levels of survivin, without affecting global translation. Knockdown of eIF4G2 reduced protein expression of the pro‐apoptotic protein Bax and increased anti‐apoptotic protein Bcl‐xL.^109^ Thus, in non‐stressed cells, the full‐length eIF4G2 was essential for maintaining cell survival by selectively promoting cap‐independent translation of pro‐survival proteins.

### 7.3 The role of eIF4G2 in cell differentiation and embryonic development

**FIGURE 5:**
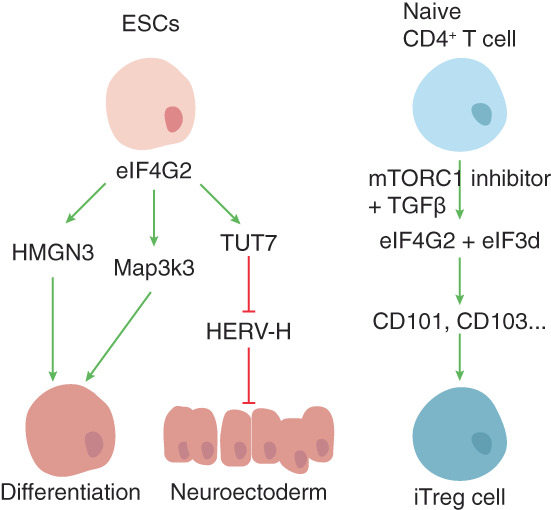
The role of eIF4G2 in the regulation of cell differentiation. In ESCs, eIF4G2 promotes cell differentiation by supporting the translation of HMGN3 and Map3k3. eIF4G2 also activates the translation of TUT7, a HERV‐H accumulation suppressor that inhibits neuroectoderm. Therefore, loss of eIF4G2 leads to aberrant HERV‐H expression, inhibiting neural differentiation. For immune cell differentiation, iTreg cells can be induced from human naive CD4+ T cells by mTORC1 inhibitor and TGF‐beta. When global translation is inhibited, eIF4G2 and eIF3d levels are increased, which supports the translation of Treg cell‐associated proteins like CD101 and CD103, thereby promoting cell differentiation.

During development and differentiation, global protein synthesis and translation of proteins in specific signalling pathways modulate cell fate^110^, ^111^ (Figure 5A). As a factor crucial to translation, eIF4G2 is critical in development. Early work established that *Eif4g2*‐null mouse embryos died during gastrulation. *Eif4g2*‐null mouse embryonic stem cells (ESCs) demonstrated normal growth but impaired differentiation induced by retinoic acid or by removing feeder cells, with defective differentiation into teratomas.^77^ A similar effect was observed in zebrafish, where eIF4G2‐ortholog knockdown embryos failed to develop mesoderm and suffered early embryonic lethality.^83^ A *Drosophila* eIF4G2‐ortholog was also studied. Loss of eIF4G2 function led to defects in several key steps of spermatid differentiation, including compact mitochondrial derivative formation and full elongation.^112^ Another work, based on isolating loss‐of‐function mutants for eIF4G2 by a reverse‐genetics approach, demonstrated that eIF4G2 was required for embryonic germband extension and metamorphosis in *Drosophila*.^113^ Together, these studies establish that eIF4G2 is essential for early embryogenesis and cellular differentiation. The function of embryonic eIF4G2 is evolutionarily conserved from nonmammalian species to mammals.

In recent years, polysome profiling and ribosome profiling, combined with mRNA abundance measurements, have identified eIF4G2 mRNA targets genome‐wide and provided critical insights into the role of eIF4G2 in differentiation. Yoffe and colleagues^93^ demonstrated that, in human ESCs, eIF4G2 knockdown by shRNA showed normal proliferation but a failure to differentiate into all lineages. The block in differentiation was not due to a global protein translational reduction, but instead it was the result of repression in the translation efficiency of eIF4G2‐regulated IRES‐driven transcripts, including the chromatin modifier HMGN3, ribosomal proteins, and transcripts involved in mitochondrial and oxidative respiration pathways. Similarly, the Yamanaka group showed that knockout of eIF4G2 reduced levels of Map3k3 and son of sevenless homologue 1 (Sos1) in mouse ESCs, suppressing the ERK signalling pathways. Forced expression of Map3k3 induced differentiation in eIF4G2‐knockout mouse ESCs.^114^ In contrast to naive pluripotent stem cells (PSCs), in which eIF4G2 depletion prevented differentiation into all cell types, eIF4G2 conditional knockout in both human and mouse primed PSCs also led to defects in neural differentiation and self‐renewal. RNA immunoprecipitation and deep sequencing (RIP‐seq) identified terminal uridylyl transferase 7 (TUT7), an RNA uridyltransferase, as an eIF4G2 target mRNA. TUT7 participated in the neural differentiation of primed PSCs by controlling human endogenous retrovirus (HERV) accumulation.^92^

In addition, eIF4G2 is also essential for immune cell differentiation (Figure 5B). iTreg cells can be induced from human naive CD4^+^ T lymphocytes by mTORC1 inhibitor and TGF‐beta. Volta et al. showed that eIF4G2 and eIF3d levels increased after induction, and eIF4G2 knockdown by siRNA impaired iTreg‐cell differentiation. This was ascribed to eIF4G2, which promoted the translation of Treg cell‐associated proteins and proteins involved in other pathways, like WNT and Sonic Hedgehog signalling.^25^

## 8 CONCLUSIONS

eIF4G2 has no binding sites for the 5′ cap binding protein eIF4E. Therefore, it has been studied with a focus on the cap‐independent translation pathway. The full‐length eIF4G2/p97, as well as the caspase‐cleavage fragment eIF4G2/p86 may support IRES‐mediated translation under stress and/or normal conditions. Recent studies, however, demonstrate that eIF4G2 also functions in translation driven by CITEs and m6A. More recently, it is shown that, even in canonical cap‐dependent translation mediated by eIF4E, eIF4G2 is also involved in leaky scanning of translated uORFs, and takes an active part in some non‐canonical cap‐dependent translation initiation.

More than two decades of research has provided a new picture in which eIF4G2 utilizes diverse translational mechanisms to regulate gene expression. eIF4G2 is more than a simple substitute for eIF4GI in translation. It also plays a critical role in physiological processes, including apoptosis, mitosis and cell survival, differentiation, and embryonic development by enhancing the translation of proteins involved in these important pathways.

## AUTHOR CONTRIBUTIONS

Yudi Liu: literature collection, figure drawing, and manuscript draft; Ji‐Fan Hu: study concept and design, manuscript revision; Jiuwei Cui, Andrew R. Hoffman and Ji‐Fan Hu: project supervision, funding acquisition; Andrew R. Hoffman: manuscript editing. All authors have read and approved the final manuscript.

## CONFLICT OF INTEREST

The authors declare that they have no competing interests.

## Data Availability

Data sharing is not applicable to this article as no new data were created or analyzed in this study.
